# Gastrointestinal Ultrasound in Infectious Diseases: A Comprehensive Review

**DOI:** 10.3390/medicina60091402

**Published:** 2024-08-27

**Authors:** Francesca Aprile, Marcello Vangeli, Mariangela Allocca, Alessandra Zilli, Marjorie Costa Argollo, Ferdinando D’amico, Tommaso Lorenzo Parigi, Silvio Danese, Federica Furfaro

**Affiliations:** 1 Department of Hepatology and Gastroenterology, ASST Grande Ospedale Metropolitano Niguarda, Piazza Ospedale Maggiore 3, 20162 Milan, Italy; francesca.aprile@ospedaleniguarda.it (F.A.); marcello.vangeli@ospedaleniguarda.it (M.V.); 2 Department of Gastroenterology and Endoscopy, IRCCS San Raffaele Hospital, Via Olgettina 60, 20132 Milan, Italy; allocca.mariangela@hsr.it (M.A.); zilli.alessandra@hsr.it (A.Z.); parigi.tommaso@hsr.it (T.L.P.); danese.silvio@hsr.it (S.D.); furfaro.federica@hsr.it (F.F.); 3 Gastroenterology Department, Federal University of São Paulo, São Paulo 04021-001, Brazil; marjorieargollo@hotmail.com; 4 Faculty of Medicine, Vita-Salute San Raffaele University, 20132 Milan, Italy

**Keywords:** infections, gastrointestinal ultrasound, bowel ultrasound, gastrointestinal diseases, infectious diseases

## Abstract

Infectious diseases affecting the gastrointestinal tract often present diagnostic challenges due to the variability in clinical manifestations and overlapping symptoms. Ultrasound imaging has emerged as a valuable tool in the assessment of gastrointestinal pathologies, offering non-invasive and real-time visualization of anatomical structures. This review aims to explore the role of ultrasound in the diagnosis and management of infectious diseases involving the gastrointestinal tract. We discuss the imaging features of various infectious etiologies, such as bacterial, viral, and parasitic infections, highlighting characteristic findings on ultrasound scans. Additionally, we provide insights into the utility of ultrasound for the assessment of treatment response. Through a comprehensive analysis of existing literature and clinical case studies, this review underscores the significance of ultrasound imaging as a frontline modality in the diagnosis and management of infectious diseases affecting the gastrointestinal tract.

## 1. Introduction

Gastrointestinal symptoms refer to a wide range of diseases, whose etiology can be complex to identify. To better understand the disease, several examinations are performed, such as computed tomography (CT) scans, stool sample analysis and colonoscopy, which can be unpleasant or invasive for patients. In this context, gastrointestinal ultrasound (GIUS) is an optimal tool as it is widely available, does not need oral or venous contrast media, provides real-time results, and can add information about extra-intestinal features such as splanchnic vessels, mesentery, omentum, and lymph nodes. The clinical applications of GIUS are numerous. It is suggested as a first examination in acute abdomen, as an initial diagnostic evaluation to detect appendicitis, acute diverticulitis, and bowel obstruction [1]. It can detect and follow up bowel involvement in inflammatory bowel diseases (IBD) and its abdominal complications; it may indicate malabsorption and celiac disease. and it may find neoplastic lesions or inflammatory masses [2]. Moreover, the role of US in infectious diseases was extensively explored, and it emerged as a meaningful tool for medical staff to better understand clinical scenarios and confirm or exclude diagnostic hypotheses [3].

This review aims to highlight the current evidence on the applications and limitations of gastrointestinal ultrasound in the context of intestinal infections. Additionally, it explores new advancements of the technique (e.g., contrast-enhanced ultrasound) which opens up new applications in the field.

## 2. Materials and Methods

### 2.1. Literature Search and Search Terms

An extensive bibliographical search was conducted in the PubMed, EMBASE, and Cochrane databases from the inception of the study to March 2024. The search strategy included medical subject heading (MeSH) terms and free-language keywords on the role of GIUS in infectious diseases.

### 2.2. Inclusion and Exclusion Criteria

The review aimed to select studies, clinical series, and cases linked to the use of GIUS and its role in diagnosis and follow-up patients with infectious diseases.

We also included English articles in which US was not the only diagnostic examination performed, but its role was relevant when supported by other results (stool examinations, blood cultures, biochemical exams, or other diagnostic examination). We excluded articles not written in English and those focused on IBD and neoplastic lesions.

## 3. Intestinal Ultrasound

### 3.1. The Technique

GIUS is a front-line investigation able to confirm or exclude diagnosis in patients with gastrointestinal symptoms. It provides specific information about the thickening and stratification of the small and large bowel wall and its vascularization. To evaluate the thickness and stratification of the bowel (using a cut-off value of 3 mm), it is essential to define the five different bowel wall layers, which include the hyperechoic lumen/mucosa interface, hypoechoic mucosa, hyperechoic submucosa, hypoechoic muscularis propria, and hyperechoic serosa. Vascularization of the bowel wall is defined using Color Doppler setting a pulse repetition frequency (PRF) of 2–5 cm/s. The presence of vessels within the wall is uncommon in a healthy bowel, but typical of inflammation or neoplastic disease. Moreover, this method explores extraintestinal organs, which are helpful for better understanding gastrointestinal clinical scenario [2]. To provide a reliable imaging examination, the quality of ultrasound scanners represents a necessary condition. The bowel ultrasound should be performed with at least two different probes. At the beginning, a low-frequency probe (2–5 MHz) is helpful for obtaining an overview of the gastrointestinal tract, due to a deeper penetration, which could be useful in obese patients or in visualizing the rectum. Later, a mid-range to high-frequency probe (5–17 MHz) is needed for a more detailed examination. However, it does not penetrate beyond 4 cm and does not allow for a proper view of deeper tissue. While standard examination does not require any specific preparation, fasting is recommended to better assess bowel motility and splanchnic vessels flow, and to avoid air filling [4,5,6].

Ultrasound imaging should follow the different parts of the bowel that are localized in specific known regions. The rectum is scanned behind the urinary bladder, but sometimes the visualization is not adequate; the sigmoid colon is in the left lower abdomen; the transverse colon could be difficult to visualize due to anatomical variability; cecum, ileocecal valve, and terminal ileum are found lying over the iliopsoas muscle in the right iliac region and iliac vessels could be used as landmarks [2]. Moreover, different segments have peculiar features: small bowel is characterized by the valvulae conniventes, which decrease in number and height from the proximal jejunum to the distal ileum. The large bowel is characterized by its haustration, which is best visible in longitudinal sections. Enlarged lymph nodes, mesentery hypertrophy, and free fluid around bowel loops should be described [2,7,8].

### 3.2. Application Fields

In a clinical setting, GIUS is routinely performed in patients with IBDs—Crohn’s disease (CD) and ulcerative colitis (UC)—to detect and follow up intestinal loops involved before and after treatment or CD complications, such as strictures, fistulas, or abscesses. Here, transabdominal bowel ultrasound showed a comparable high sensitivity to magnetic resonance enterography (MRE) [9,10]. Stratification, thickening of the bowel wall, and assessment of wall layers define active disease [11].

In emergency care, bowel ultrasound plays an active role, and it is often suggested as the first imaging tool in patients with acute abdomen [2].

Moreover, signs of malabsorption and celiac disease (mesenteric lymphadenopathy, dilated small bowel loops with increased fluid content and increased peristalsis) could be detected in patients with abdominal symptoms and variations in bowel changes [2].

## 4. GIUS in Infectious Disease

Various parasites, bacteria, and viruses are responsible for gastrointestinal infections that result in similar symptoms such as abdominal pain, diarrhea (with or without blood), and fever. Stool cultures, parasitic examinations, fecal calprotectin, CRP, ESR, and blood count are considered first-level examinations and may be sufficient for diagnosis in most infectious enterocolitis. However, US imaging can add relevant information and help in differential diagnosis. Here, we described different infectious diseases that determine the small and large bowel involvement, in which bowel ultrasound can contribute relevant findings.

### 4.1. Parasites

#### 4.1.1. Anisakiasis

Anisakiasis is an infectious disease due to the ingestion of raw or undercooked fish (mostly hake, anchovies, bonitos, mackerel, salmon, and squid) that contains Anisakis larvae. It is most common in Asia, but an increasing number of cases are diagnosed in Europe due to the diffusion of raw food eating habits [12,13].

This parasite can affect all the gastrointestinal sites from the stomach to the colon by adhering to or penetrating the mucosa. Unfortunately, the disease presents with non-specific symptoms or signs, making it difficult to diagnose. Abdominal pain is very frequent, and its localization depends on which part of the digestive system is affected. If the stomach is affected, patients may experience acute gastric pain, nausea, and vomiting within a few hours of ingestion. However, if the small intestine is involved, abdominal pain is more common in the right iliac fossa. Neither fever nor diarrhea is a common symptom, and laboratory exams show a variable leukocyte count which does not reveal a specific picture.

Gastric anisakiasis is diagnosed via a radiological or endoscopic examination that shows the whole worm. Whereas, when the parasite involves intestinal sites, the diagnosis is more demanding. Since the patients may develop an acute abdomen, surgery exploration is often required.

An old Japanese study explored the role of US in diagnosing anisakiasis. The study evaluated 18 patients who had a confirmed diagnosis of anisakiasis between 1985 and 1990. All the patients had a history of eating raw fish, abdominal pain, segmental thickening of the bowel walls, and antibodies to anisakiasis. US showed a thickening of the intestinal wall, ranging from 5 to 15 mm. Terminal ileum was the most common site, while the proximal ileum and jejunum were less frequently involved. Fluid retention within the loops and free peritoneal fluid was a common finding, whose cytologic examination revealed eosinophilic infiltration. Additionally, edematous Kerckring folds and decreased peristalsis were present. Oral barium tests confirmed irregular luminal narrowing of the small bowel in all patients, with edematous folds observed, but no findings of ulceration. The patients underwent conservative treatment, and a correct diagnosis prevented them from undergoing unnecessary surgery. Symptoms and ultrasound findings returned to normal after one week [14].

In 2022, Fornell Pérez et al. reviewed imaging findings in anisakiasis, highlighting the key role of US in identifying unsuspected cases and in ruling out different diagnoses. Common findings were circumferential wall thickening with active vascularization in Color Doppler. Stratification was usually preserved with mucosal/submucosal edema and edema of the valvulae conniventes. Moreover, proximal intestinal dilation with reduced motility of affected loops and ascites was also found [15].

In gastric involvement, a diffuse submucosal edema and wall thickening associated with ascites were shown [16]. Furthermore, GIUS helps to avoid the misdiagnosis of anisakiasis with other diseases. Usually, the presence of ascites and involvement of perigastric tissue help to differentiate it from acute gastritis, hypertrophic gastritis, or Ménétrier disease. Eosinophilic gastroenteritis is characterized by segmental wall thickening, which is caused by submucosal edema. Additionally, this condition may result in concentric luminal stenosis, dysmotility, and persistent signs and symptoms.

#### 4.1.2. *Giardia lamblia*

*Giardia lamblia* is the most common protozoan parasite in developing countries, as well as in the United States [17]. It frequently causes traveler’s diarrhea without blood and mucus, steatorrhea, flatulence, and bloating by ingestion of food or water that is contaminated by cysts [18]. After contaminated food is consumed, Giardia cysts can transform into their active trophozoite form due to the combination of low stomach acidity and pancreatic enzymes that are released in the duodenum. After trophozoites divide, they can attach firmly to the intestinal cells without invading them, and cause apoptosis of enterocytes, dysfunction in the intestinal barrier and host response, brush border microvilli shortening, and faster GI transit time [19]. Encystation occurs as the parasites transit toward the colon due to different pH and are eliminated through the feces. Diagnosis mainly consists of identifying *G. lamblia* cysts in fecal smears of three pooled individual specimens, but antigen detection in stool samples using immunofluorescence could also be performed [20,21]. In difficult cases biopsy specimens or other biological samples, such as duodenal aspirates, could help [22,23]. The management of patients is based on the use of metronidazole.

In 2007, Njemanze et al. conducted a study to investigate the potential of high-frequency bowel ultrasound in detecting intestinal alterations in patients infected with *G. lamblia.* The study involved a comparison of 100 symptomatic giardiasis patients, 40 symptomatic amebiasis patients, and 40 healthy controls. All participants underwent US with and without water contrast, and the researchers noted significant wall thickening (6.3 ± 1.3 mm in the duodenum, 8.8 ± 1.4 mm in the ascending colon; 9.2 ± 1.2 mm in the descending colon), flattening of Kerckring folds, and/or colonic haustrations in the giardiasis group.

Also, bowel wall stratification was modified: the duodenal and colon wall became more hyperechoic with poor distinction of different layers and triband structure is replaced by a single hyperechoic band. Moreover, hyperechoic floating foci (HFF) were observed as small echogenic particles in floatation in all directions between peristaltic waves aiding sonographic real-time imaging of their chaotic movements. It was observed in 100% of duodenal cases and heavy colon infections. Last but not least, US revealed asymmetric colonic contractions and pseudo-haustrations that may derive from herniation of the submucosa through the mucosal surface. The results suggest that high-frequency US could be an effective diagnostic tool in identifying intestinal changes associated with *G. lamblia* infection [24].

#### 4.1.3. *Schistosoma mansoni*

Schistosomiasis is a parasitic infection caused by trematodes from the *Schistosoma* genus. This infection enters the human host through the skin via cercariae. These parasites then travel through the veins to the lungs, heart, and ultimately the liver. The mature worms exit the liver through the portal vein system and reside in the mesenteric venules. The location of these venules varies depending on the species. For instance, *S. japonicum* is usually found in the superior mesenteric vein that drains the small intestine, while *S. mansoni* is more commonly found in the inferior mesenteric vein that drains the large intestine. There is no clear consensus on the effectiveness of bowel ultrasound in this context, and the results are still being debated. In 1994, Dittrich et al. performed bowel ultrasound on patients infected by *Schistosoma mansoni* in an endemic area in Senegal. Thickened intestinal wall was present in all patients and it appeared as a pseudokidney sign in the longitudinal section. This was characterized by an echoless area corresponding to the intestinal wall and a highly echogenic inner structures corresponding to bowel content. In the transverse sections, the same phenomenon formed a circular or cocardic structure. Other relevant findings were the high echogenicity of the mucosa and hyperchoic mesenteric structures. Conversely, in 2018, Tamarozzi et al. performed a prospective case–control study on intestinal unenhanced US using a high-frequency probe to assess *S. mansoni* outside endemic areas. Bowel US was performed on 17 patients with a diagnosis of *S. mansoni* infection, confirmed by microscopy and/or PCR, one month after medical treatment with praziquantel. The study showed no significant difference in wall thickness in any of the bowel segments analyzed (from terminal ileum to sigma) in the study and control group. Both studies did not identify polyps in the bowel which are a common endoscopic finding [25,26,27].

Several reasons could explain the opposite results obtained in the two studies. These include factors such as whether the study was conducted in an endemic or non-endemic area, whether children were included or excluded in the study, differences in the intensity of infection between the two cohorts, variations in the diagnostic method used to detect parasites in the stool samples, variations in the prevalence and types of intestinal infections, and differences in the image resolution of the ultrasound machines and probes used. Dittrich and colleagues used a convex low-frequency probe, while the Italian study used a linear high-frequency probe.

### 4.2. Bacteria

#### 4.2.1. *Salmonella typhi*

There are various serovars of Salmonella enterica, with *S. typhi* being the most dangerous due to its association with typhoid fever. This bacteria can invade the intestine, leading to an infection that is initially asymptomatic, but it may result in systemic dissemination and a transient primary bacteremia. Unlike most diseases caused by nontyphoidal Salmonella (NTS) serovars, *S. typhi* does not cause rapid inflammation or diarrhea [28]. During the first week, symptoms of the disease include a stepwise increase in body temperature. In the second week, abdominal pain and red or salmon-colored macules on the abdomen and trunk may appear. In the third week, the disease can cause hepatomegaly and splenomegaly, intestinal bleeding, and perforation due to lymphatic hyperplasia in the Peyer’s patches of the ileocecal region. In severe cases, secondary bacterial infections can occur, leading to bacteremia and peritonitis. In some cases, the patient may develop septic shock or an altered state of consciousness. Blood cultures are positive in 50 to 70 percent of patients with typhoid [29]. The diagnosis can also be made with culture of stool, urine, rose spots, or duodenal contents. The Widal test is of limited clinical utility in endemic areas as positive results may represent previous infections since it detects anti-*S. Typhi* antibodies.

To explore the role of US in patients with an ascertained diagnosis of typhoid fever, Younis identified the main echographic features within one week of symptom onset. A total of 350 patients underwent abdominal and bowel ultrasound. The most common findings were hepatomegaly in 110 out of 350 patients (31.4%), prominent bile ducts in 227 out of 350 patients (64.85%), splenomegaly in all 350 patients (100%), mesenteric lymphadenopathy in 150 out of 350 patients (42.85%), bowel wall thickening in 125 out of 350 patients (35.71%), acalculous cholecystitis in 57 out of 350 patients (16.28%), perforation in 4 out of 350 patients (1.14%; 3 ileal and 1 gallbladder), and abscess in 12 out of 350 patients (3.42%). The study concluded that although there are no specific ultrasound signs for typhoid fever, these findings are typical enough to start treatment in patients with negative cultures. Conversely, a normal US can rule out the diagnosis of typhoid fever [30].

#### 4.2.2. Salmonella and *Campylobacter jejuni*

**Figure 1:**
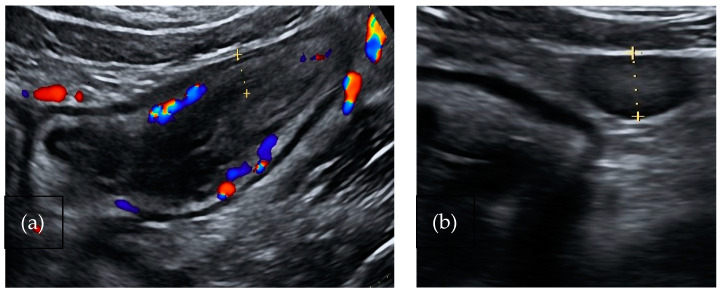
A 37-year-old-man affected by Campylobacter infection. (**a**). Bowel ultrasound shows thickening of the right colon wall (5.8 mm) with active inflammation (color flow Doppler signals in both the bowel wall and surrounding mesenteric fat). (**b**). Presence of lymphadenopathy.

Salmonella and Campylobacter are Gram-negative bacteria responsible for self-limiting ileocecal infections. The typical involvement of terminal ileum and caecum determines acute right lower abdominal pain (Figure 1a,b). Diarrhea is absent or mild and stool cultures are not always requested; therefore, diagnosis is delayed. These symptoms are frequently considered as clinical signs of appendicitis and often lead to an unnecessary appendicectomy. US findings include marked hyperechoic thickening of the mucosa and submucosa of the terminal ileum and caecum with or without lymphadenopathy. Mesenteric fat and the appendix are not involved [31].

#### 4.2.3. Yersinia

*Yersinia enterocolitica* (Ye) is the most common species that infects human beings, with an estimated prevalence of about 1% among bacterial enteritides [32]. It invades the lymphoid follicles of Peyer’s patches in the small intestine and usually causes enteritis with mesenteric lymphadenitis and terminal ileitis. The location makes it difficult to distinguish from acute appendicitis, terminal ileitis, and mesenteric lymphadenitis and may even mimic a fistulizing Crohn’s disease of the ileo-cecal region.

An interesting Japanese study compared clinical features and ultrasonographic findings between Ye and other bacterial enteritides diagnosed from stool cultures. The study included 27 patients with Ye and 29 patients with other bacterial enteritides, including 14 cases of C. jejuni enteritis, 13 cases of Salmonella, and 2 cases of *E. coli*.

No significant difference was found in clinical symptoms or signs between the two groups of patients. However, bowel US confirmed that patients with Ye had ileocecal lymph nodes (ICLNs) with a more rounded shape and it revealed a higher frequency of pericecal hyperechoic region development. This is an extraluminal hyperechoic band that is distinguishable from the terminal ileum and is similar to the “creeping fat” frequently observed in CD. They concluded that a major–minor axis ratio of less than 1.51 or the presence of a pericecal hyperechoic region is 100% sensitive in differentiating Ye enteritis from other enteritis. The combined presence of a mean ICLN major–minor axis ratio of less than 1.51 and the presence of a pericecal hyperechoic region had a specificity of 100%. Moreover, all cases with a pericecal hyperechoic region displayed a spontaneous resolution of this finding within a few weeks after starting the appropriate treatment [33].

#### 4.2.4. *Mycobacterium tubercolosis*

Tuberculosis (TB) is caused by *Mycobacterium tuberculosis,* which mainly affects the lungs. However, it can also lead to extra-pulmonary diseases, including abdominal involvement in 11–12% of cases. Despite being preventable and usually curable, TB remained the world’s second leading cause of death from a single infectious agent in 2022, after SARS-CoV-2, and caused almost twice as many deaths as human immunodeficiency virus (HIV) and acquired immunodeficiency syndrome (AIDS). About a quarter of the global population is estimated to have been infected with TB [34]. Following the infection, the risk of developing TB disease is highest in the first 2 years (approximately 5%), after which it is much lower. Early diagnosis and specific treatment are essential to decrease the high death rate (about 50%) associated with untreated TB disease. The symptoms of intestinal tuberculosis (ITB) are not specific, which makes it difficult to differentiate from other intestinal diseases. Any part of the gastrointestinal tract can be affected, but the most affected sites are the ileocecal region (60–70%), colon, and jejunum. Isolated involvement of the colon is seen in about 10% of the cases. Anal tuberculosis is a rare condition that can sometimes cause multiple fistulae, which can resemble CD. The three main forms of ITB described in the literature are ulcerative, hypertrophic or ulcero-hypertrophic and stricturing [35]. The ulcerative form is the most common pattern and usually presents with superficial transverse ulcers. The hypertrophic form presents as a hyperplastic reaction around the ulcer that often results in inflammatory mass. The stricturing form represents fibrosis and presents as a stricture. Intestinal obstruction is a common complication caused by strictures, and intestinal perforation usually occurs proximal to the site of the stricture [36]. Less common complications include intussusception in hypertrophic cases, as well as abscess or fistulization [37]. Only histology may distinguish intestinal tuberculosis from other intestinal diseases: it is highly specific for tuberculosis with a specificity of over 95%, which shows confluent granulomas with caseation necrosis and histiocyte-lined ulcers. However, the sensitivity of histology is limited to less than 50% [38,39]. Imaging can provide crucial information regarding the patient’s disease. The most significant US findings are ascites (56%), retroperitoneal and mesenteric lymphadenopathy, usually with a node diameter greater than 1.5 cm (18%), and intestinal involvement (8%). Other less common ultrasound findings include mesenteric abscesses, thickened omentum, intramural abscesses, fistulas, and mesenteric thickening [40,41]. In 2015, Yang et al. conducted a retrospective study to examine the ultrasound features of ITB in 31 patients whose diagnosis was confirmed through histological analysis. The patients underwent standard abdominal US and contrast-enhanced ultrasound (CEUS) to evaluate the pattern of bowel wall enhancement. Out of the 31 patients, 27 had ileocecal involvement with a hypoechoic pattern, and the mean thickening of the bowel wall was 1.38 cm, ranging from 0.56 to 2.20 cm. Contrast media shows faster enhancement in thickened bowel walls due to abundant granulomas with newborn capillaries and hemangiectasis induced by regional inflammation. There were two different patterns of enhancement. In type 1 enhancement (4/31 patients), the serosa was quickly enhanced first, followed by gradual enhancement of the mucosa. In the remaining 27 patients, the entire bowel wall was quickly diffusely enhanced (type 2 enhancement). Histopathologically, type 1 showed lower levels of bowel wall damage and a fewer number of granulomas. Moreover, the enhancement of the thickened bowel wall was homogeneous in nine patients, while the remaining patients showed inhomogeneous enhancement, which correlated with caseous necrosis. All patients with TB in the ileocecal region had enlarged lymph nodes with destroyed lymph node hilum and hypo-to-anechoic central lymph node areas, corresponding to caseous necrosis [42]. Moreover, bowel ultrasound is also accurate in the evaluation of the response in patients with ITB [43].

##### Special Categories

Ultrasound findings are not sufficient to distinguish ITB from CD, which are both chronic granulomatous diseases with striking similarities in clinical, endoscopic, imaging and histological findings [44,45,46]. When a specific treatment for CD does not yield an adequate response, reconsidering the diagnosis is appropriate. Retesting for TB, repeating a CT scan and a colonoscopy with terminal ileum intubation are possible strategies [46]. Moreover, TB screening is mandatory in antiTNF IBD candidates, and patients with risk factors for TB could develop the disease with a more frequent intestinal involvement, even after a negative screening. TB test results should be carefully evaluated in patients under treatment with steroids or immunosuppressants and in those who are at high risk of TB (e.g., patients with hemodialysis or organ transplantation, patients with HIV, prisoners, health care workers, immigrants or travelers from countries at risk, the homeless, drug users, and elderly persons) [47,48]. Systemic features such as fatigue and low fever with an epidemiological history should raise the hypothesis of TB and lead to a prompt guided investigation. Additionally, a hypoechoic appearance of bowel wall with extraintestinal features such as splenomegaly are highly suggestive of TB.

Moreover, in HIV-positive individuals for whom abdominal tuberculosis is more common, abdominal US appears to have 63% sensitivity and 68% specificity. The low sensitivity of abdominal ultrasound means clinicians should not use a negative test result to rule out the disease, but rather consider the result in combination with other diagnostic strategies. In conclusion, bowel ultrasound could represent in ITB just a piece of the puzzle that helps clinicians to make the right diagnosis.

##### *Clostridium difficile* 

**Figure 2:**
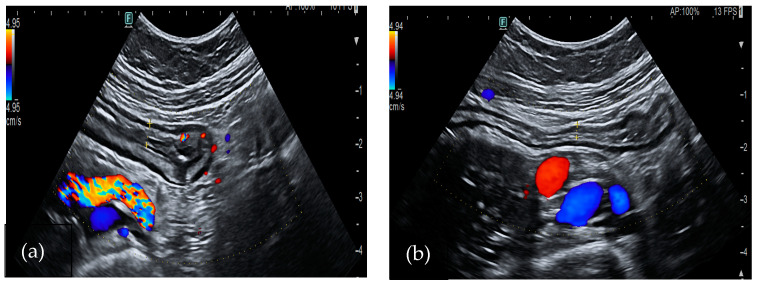
A 36 year-old-woman with Clostridium diarrhea. Bowel ultrasound showed colon wall thickening (**a**) that normalized after adequate treatment (**b**).

*Clostridium difficile* (CD) is an anaerobic, Gram-positive, spore-forming bacillus that represents a leading cause of antibiotic-associated diarrhea. It produces two main exotoxins, toxin A and toxin B, which can harm colon epithelium and trigger an inflammatory reaction. It may lead to mucosal necrosis and the formation of pseudomembranes, which consist of an inflammatory exudate covering the exposed mucosa [49,50]. The diagnosis of the disease consists of the detection of toxins in the stool, but radiological exams also supply useful information about the infection. In particular, a CT scan is the most sensitive examination to define colon extension. In most cases, it shows pancolitis with mural thickening, even if infections limited to focal parts of the colon have also been described [51]. Moreover, a CT scan may also reveal contrast material appearing between pseudomembranes and ascites, known as the “accordion sign”. In bowel ultrasound, the wall of the large bowel appears thickened between 6 and 20 mm (Figure 2). It often shows submucosal and mucosal edema, which is represented by a heterogeneous intermediate-echogenicity band, with preservation of the muscular layer [52]. Moreover, suppurating ulcers may appear as hypoechoic defects in the hyperechoic interface between mucosa and lumen. In severe cases coalescent pseudomembranes appear as linear echogenic structures parallel to the mucosal interface [53]. Ascites is also present more commonly than in IBD, which is probably related to hypoalbuminemia and increased bowel permeability. In 2015, a case–control study compared the presence of five ultrasonographic features (colon thickness, internal and external ring, large bowel content diminution, and ascites) in patients who were hospitalized with diarrhea. The study group was toxin-positive, while the control group was toxin-negative. The colonic wall thickening was present in 91% (*n* = 61) of patients with CD toxin, while it was present in only 21% (*n* = 15) of control group. This feature had high negative and positive predictive values for *Clostridium difficile* infection (CDI), and the authors concluded it may help in confirming or excluding the diagnosis of CDI. Furthermore, the other ultrasound parameters had high positive predictive values and may help to confirm CDI [54]. Therefore, even if none of these findings are pathognomonic, performing bowel ultrasound is a useful tool in an acute setting, especially in patients with kidney disfunction.

### 4.3. Virus

#### Cytomegalovirus

**Figure 3:**
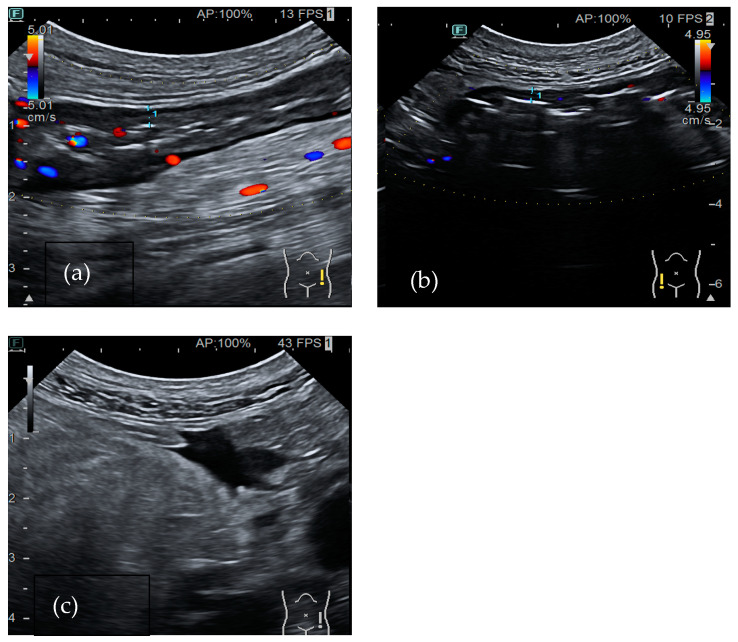
A 76-year-old-male patient was diagnosed with long-term long-term left ulcerative colitis. A bowel ultrasound was performed after the onset of bloody diarrhea during mesalamine treatment. US showed diffuse colonic wall thickening: (**a**) 4.7 mm in the descending colon and (**b**) 3.6 mm in the right colon. Active vascularization (**a**) with free fluid (**c**) was also found. The biopsies performed during colonoscopy revealed a CMV infection.

Cytomegalovirus (CMV) typically causes pancolitis in immunosuppressed patients. Moreover, in adults with UC on steroid treatment, superinfection with CMV should be suspected, especially in patients who are resistant to glucocorticoids (Figure 3). Diagnosis requires a high level of suspicion since colonoscopy biopsies are necessary [55]. Bowel ultrasound shows wall thickening and pericolic fat stranding, occasionally accompanied by free fluid. Lymph nodes enlargement is not common. These US findings are not enough to establish a definite diagnosis and IBD should be ruled out [3].

## 5. Conclusions

In this review, we explored a wide range of applications of bowel ultrasound in the context of infectious disease. This technique is a cost-effective, highly reproducible, and non-invasive tool that offers relevant information to clinicians in real time. Intestinal and extraintestinal findings can assist in diagnosing and promptly initiating appropriate treatment (Table 1). Markable ultrasound features of bowel wall thickness and stratification, presence of splenomegaly, enlarged lymph nodes with a necrotic content, and other findings, could suggest a specific etiology.

Moreover, this specific non-invasive low-cost tool could impact daily practice in emerging countries where infectious diseases are very common, especially tuberculosis, and where cost-effective strategies are vital to better assist and monitor patients under treatment. This could drastically impact the acute setting of cases presenting with diarrhea and or abdominal pain, allowing for a more invasive approach to specific cases. However, US alone is insufficient for diagnosis, as many infectious diseases have similar imaging features. Furthermore, the presence of air and limited experience can affect accurate diagnosis. The diagnostic performance of bowel ultrasound in this setting will be enhanced by improvements in training, high-performance of machines and probes, and shared protocols.

## Figures and Tables

**Table 1:** Ultrasound features in infectious disease: similarities and differences.

Infectious Disease		Location	US Feature
Parasites	*Anisakiasis*	Stomach Ileum/ileocaecal region	Thickening of the stomach/intestinal wall Edematous Kerckring folds Reduced peristalsis Ascites with eosinophilic infiltration
	*G. lamblia*	Duodenum Colon	Thickening of the intestinal wall (hyperechoic pattern) Flattened Kerckring folds Flattened colonic haustrations Fluid-filled lumen (hyperechoic floating foci) Asymmetric colonic contractions
	*Schistosoma mansoni*	From terminal ileum to sigma	Thickening of the intestinal wall
Bacteria	*Campylobacter jejuni*	Ileum/ileocaecal region	Thickening of the intestinal wall Lymphoadenopathy
	*Salmonella enterica*	Ileum/ileocaecal region	Thickening of intestinal wall
	*Salmonella typhi*	Ileum/ileocaecal region	Thickening of intestinal wall (37%) Splenomegaly (100%) Prominent bile ducts (64%) Hepatomegaly (31%) Acalcolous cholecystitis (16%)
	*Yersinia enterocolitica*	Ileum/ileocaecal region	Pericecal hyperechoic region Ileocecal lymph nodes with a more rounded shape
	*Mycobacterium tubercolosis*	Ileum/ileocaecal region	Thickening of the intestinal wall (hypoechoic pattern) Ascites Retroperitoneal and mesenteric lymphadenopathy
Virus	*Cytomegalovirus*	Colon	Thickening of the intestinal wall Ascites
	Pseudomembranous colitis (*Clostridium difficile*)	Colon	Thickening of the intestinal wall Hypoechoic defects in the hyperechoic interface between mucosa and lumen (ulcers) Linear echogenic structures parallel to the mucosal interface (coalescent pseudomembranes in severe cases) Ascites

## Data Availability

Data sharing not applicable.

## Abbreviations

CT	computed tomography
GIUS	gastrointestinal ultrasound
IBD	inflammatory bowel disease
PRF	pulse repetition frequency
MeSH	Medical subject heading
CD	Crohn’s disease
UC	ulcerative Colitis
MRE	magnetic resonance enterography
HFF	hyperechoic floating foci
NTS	nontyphoidal salmonella
Ye	*Yersinia enterocolitica*
ICLNs	ileocecal lymph nodes
TB	tuberculosis
HIV	human immunodeficiency virus
AIDS	acquired immunodeficiency syndrome
ITB	intestinal tuberculosis
CEUS	contrast-enhanced ultrasound
ATT	anti-tubercular therapy
CD	clostridium difficile
CDI	clostridium difficile infection
CMV	cytomegalovirus
